# Mother's care-seeking behavior for neonatal danger signs from qualified providers in rural Bangladesh: A generalized structural equation modeling and mediation analysis

**DOI:** 10.3389/fped.2022.929157

**Published:** 2023-01-04

**Authors:** Rashidul Azad, Sk Masum Billah, Bal Ram Bhui, Nazia Binte Ali, Samantha Herrera, Joseph de Graft-Johnson, Lyndsey Garg, Sabrina Sharmin Priyanka, Shams Zubair, S. M. Rokonuzzaman, Mohammad Mahmoodur Rahman, Umme Salma Jahan Meena, Shams El Arifeen

**Affiliations:** ^1^ International Centre for Diarrhoeal Disease Research, Bangladesh (Icddr,b), Dhaka, Bangladesh; ^2^ The University of Sydney School of Public Health, Sydney, NSW, Australia; ^3^ Save the Children, Dhaka, Bangladesh; ^4^ Harvard T.H. Chan School of Public Health, Boston, United States; ^5^ PATH, Washington, DC, United States; ^6^ Save the Children, Washington, DC, United States

**Keywords:** care-seeking behavior, neonatal danger signs, neonatal illness, newborn illness, Bangladesh, generalized structural equation modeling (GSEM), mediation analysis

## Abstract

**Background:**

Neonatal deaths contribute to nearly half (47%) of under-five mortality globally and 67% in Bangladesh. Despite high neonatal mortality, care-seeking from qualified providers for newborn danger signs remains low. Identification of direct and indirect factors and their pathways affecting care-seeking will help to design a well-targeted intervention. This study assessed the direct, indirect, and total effect of the predictive factors on neonatal care-seeking in Bangladesh.

**Materials and methods:**

This was a cross-sectional baseline household survey conducted in 14 districts of Bangladesh in 2019 with 17,251 recently delivered women (RDW) with a live birth outcome in the preceding 15 months. We used a two-stage stratified cluster sampling process to select the samples from 14 districts. We investigated the inter-relationship of maternal background characteristics, maternal health utilizations, child/neonate factors, health service delivery-related factors and newborn danger sign knowledge with newborn care-seeking practices and estimated the direct, indirect, and total effects using Generalized Structural Equation Modeling (GSEM) and mediation analysis. *p*-value = 0.05 was considered statistically significant. The result of the mediation analysis was reported in Log Odds (LOD). The positive LOD (LOD > 0) implies a positive association.

**Results:**

Half of the mothers (50.8%) reported a neonatal illness and among them, only 36.5% mothers of sick neonates sought care from qualified providers. Our mediation analysis showed that maternal health utilization factors, i.e., 4 + antenatal care visits (ANC) from a qualified provider (LOD: 0.63, 95% CI: 0.49, 0.78), facility delivery (LOD: 0.74, 95% CI: 0.30, 1.17) and postnatal care (PNC) from a qualified provider (LOD: 0.50, 95% CI: 0.21, 0.78) showed the highest total effect over other factors domains, and therefore, were the most important modifiable predictors for qualified neonatal care-seeking. Other important factors that directly and/or indirectly increased the chance of newborn care-seeking from qualified providers were household wealth (LOD: 0.86, 95% CI: 0.70, 1.02), maternal education (LOD: 0.48, 95% CI: 0.32, 0.63), distance to nearest health facility (LOD: 0.20, 95% CI: 0.10, 0.30), community health worker's (CHWs) home visits during ANC (LOD: 0.24, 95% CI: 0.13, 0.36), neonatal danger sign counseling after delivery (LOD: 0.20, 95% CI: 0.06, 0.34) and women's knowledge of neonatal danger signs (LOD: 0.37, 95% CI: 0.09, 0.64).

**Conclusion:**

The inter-relationship and highest summative effect of ANC, facility delivery, and PNC on newborn care-seeking suggested the maternal care continuum altogether from ANC to facility delivery and PNC to improve care-seeking for the sick newborn. Additionally, referral training for unqualified providers, targeted intervention for poorer households, increasing CHWs home visits and neonatal danger sign counseling at the facility and community should also be considered.

## Introduction

Globally, tremendous progress in child survival has been made over the last three decades. Under-five child deaths decreased by 58 percent between 1990 and 2018, from 12.5 million to 5.3 million. Almost half (2.5 million) of these deaths were classified as neonatal deaths, occurring in the first 28 days of life, showing the high burden of child mortality at this early age (1).

Neonatal mortality varies greatly across geographical regions and is mostly concentrated in low and middle-income countries. In Sub-Saharan Africa and Southern Asia, the average neonatal mortality rate (NMR) ranges between 25 and 28 per 1,000 live births, whereas in Europe, Northern America, Oceania, and Eastern Asia the NMR ranges between 2 and 4 per 1,000 live births (1). In Southern Asia, between 2015 and 2018, almost 1 million newborns died each year, with the neonatal death proportion among under-five child death increasing from 46% in 1990 to 62% in 2018, which is the highest proportion of deaths in South Asia compared to other regions of the world (1). For Bangladesh, NMR has increased to 30 in 2018 from 28 in 2014; which led to an increase of neonatal death proportion among under-five child deaths from 61% to 67% during the same period (2, 3).

A neonatal death surveillance in four districts of Bangladesh in 2016 reported that the leading cause of neonatal death was birth asphyxia (43%), followed by infections (29.3%), and prematurity (22.2%). Day-wise disaggregation showed that 63.4% of newborns who died on their birthday died due to asphyxia, whereas infections were the main cause of neonatal deaths among newborns who died between days 1–6 (48.8%) and days 7–28 (57.5%) (4). Other studies in Bangladesh and similar contexts reported similar causes and trends (5–7). Early identification, treatment and/or referral of these major causes of newborn deaths are essential to reduce neonatal deaths, especially infections, which are better managed by a qualified provider when identified and referred early (8–10).

In low and middle-income countries (LMICs), several interventions implemented around the time of birth have been proven effective in reducing neonatal mortality. These interventions are delivered through an integrated community and health facility platform (8, 11, 12). Domiciliary services by health workers' home visits, birth preparedness education, newborn care practices, identification of newborn danger signs, and early care-seeking have been proven effective to reduce neonatal mortality (8, 11–14). However, caregivers' timely identification of neonatal danger signs and subsequent care-seeking from qualified health providers should be the core of a successful healthcare intervention for neonatal survival (14).

Care-seeking behavior for sick newborns remains low and is a key challenge to reduce neonatal deaths in LMICs (14). This is further compounded by slow recognition of danger signs and delayed care-seeking, as the health condition of sick newborns declines rapidly and can quickly progress to death (15, 16). Care-seeking behavior is a complex decision-making process (17). Multiple factors (i.e., illness severity perception, maternal and newborn factors, health care utilization practices, socio-economic and cultural factors, geographical location, quality of care, cost of care, etc.) influence the caregivers' and family members' care-seeking behavior for neonatal illness (14, 18–21).

In-depth understanding of care-seeking behaviors for newborns with danger signs would help to design effective interventions at both community and facility levels (19, 22). There are limited data and studies on newborn illness care-seeking practices in Bangladesh. Earlier studies in Bangladesh investigated the direct relationship of predictor variables with care-seeking practices but did not explore the inter-relationship among the predictor variables (21, 23–26). The most important or influential predictor variables on neonatal care-seeking behavior can be identified through analysis of the inter-relationships among predictor variables and their association with the main outcome variable. This nuanced understanding of important predictors of care-seeking can be used by policy makers, program managers and researchers to inform the design and testing of interventions to improve care-seeking behavior for sick newborns.

Structural Equation Modeling (SEM) has been widely used to simultaneously estimate and test the effects of variables and their inter-relationships within the complex hypothetical conceptual framework (27–30). It allows identification of the pathways of cause and effect from distal factors to proximate factors and to the outcome of interest. Generalized Structural Equation Modeling (GSEM) is the extended and generalized form of SEM for the estimation and testing the effects of categorical, continuous and count variables (31). We used GSEM to explain the relationships among predictor variables in determining mothers' care-seeking behavior. We also conducted a mediation analysis to estimate the direct, indirect (mediated), and total effects of predictors on the main outcome among each possible mediated pathway found significant in the GSEM analysis (32, 33).

We include the description of the hypothesized conceptual/theoretical model of the inter-relationships between the predictors and the neonatal care-seeking outcome in the next section. Our research objective is to examine this hypothetical inter-relationship with GSEM and identify the most important predictors that have both direct and indirect relationships among them and with the outcome variable. We also estimated the direct, indirect (mediated), and total effect of the predictor variables on neonatal care-seeking.

## Materials and methods

### Study design, setting, source population and study population

This was a population-based cross-sectional baseline survey conducted in 14 districts (Noakhali, Feni, Chandpur, Lakshmipur, Brahmanbaria, Habiganj, Manikganj, Faridpur, Madaripur, Kushtia, Kishoreganj, Natore, Rajbari and Bhola) that are part of the evaluation of MaMoni Maternal and Newborn Care Strengthening Project (MNCSP) and included both intervention and comparison districts. The MaMoni MNCSP project was implemented in 10 intervention districts out of 64 districts in Bangladesh. MaMoni MNCSP was a United States Agency for International Development-funded project (2018–2023) focused on strengthening public sector maternal and newborn care (MNC) services through increased and equitable accessibility and utilization of quality MNC services in Bangladesh. The key intermediary objectives of MaMoni MNCSP were to advance health system responsiveness, improve MNC service quality and governance, access and demand for services and healthy household practices, and strengthen national capacity to deliver these services with quality at scale. MaMoni MNCSP was implemented by a consortium of local and international partners led by Save the Children. Details of project interventions, project evaluation design, and study settings are available elsewhere (35). As a MaMoni project consortium member, icddr,b conducted the cross-sectional baseline household survey to measure population level coverage of key maternal and newborn practice indicators. The source population was the recently delivered women (RDW) who gave birth within the last 15 months irrespective of birth outcome status in the above-mentioned 14 districts. The study population of RDW was selected from the source population with the sampling techniques described below.

### Sample size, sampling and study subjects

The sample size was primarily calculated to detect the minimum difference between intervention and comparison districts considering neonatal mortality as the main outcome of the MaMoni MNCSP evaluation study. The neonatal mortality rate was 38 per 1,000 life birth (2) and during the sample size calculation; we assumed a 25% reduction of neonatal mortality as per the intervention effect. Considering 2.5:1 intervention to comparison samples size ratio, 80% power, 5% level of significance, 1.5 design effect, and 5% non-response, the calculated sample size was 16,654 in total (11,896 in intervention districts and 4,758 in comparison districts). Secondarily, we also calculated the sample size to estimate the district level coverage of public health facility delivery. Considering 14.3% of public facility delivery prevalence (2), 2.5% absolute precision, 5% non-response and 1.5 design effect, the calculated sample size for each district was ≅1200. So, the total sample size for the 14 evaluation districts was 16,800.

We followed a two-stage stratified cluster sampling process for selecting 1,200 samples from each district. We selected 120 clusters per district and each cluster needed to comprise 10 samples (RDW). Considering the average household size 4.4 and crude birth rate of 21.0 per 1,000 population per year (2), we needed to list at least 120 households to attain ∼10 samples. We conducted the village level clustering and followed the Probability proportional to size (PPS) sampling for the selection of cluster/village within each district. Considering the time and cost involved in a complete listing of households of villages, the project decided not to do a complete listing of all households in each selected village. Alternatively, we selected a section of a village with 120 households. Therefore, we needed a starting point to start listing 120 households for each cluster within the selected village. We used the eligible couple registers maintained by the Family Welfare Assistants (FWA) to randomly select the starting household (index household) to determine starting household. From that index household, we continued till we reach 120 households to complete one cluster. From each cluster we expected to find 10 RDW samples; however, if there were more RDWs we interviewed all.

**Figure 1:**
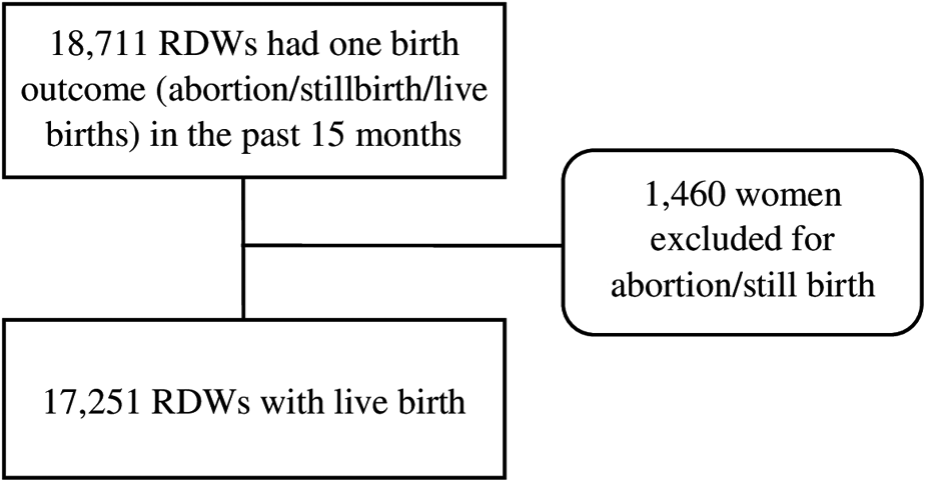
Selection of the sample for the analysis.

A total of 18,711 RDW having at least one birth outcome (abortion/stillbirth/live birth) in the past 15 months were interviewed as the study population in the survey. For the analysis, 1,460 women were excluded who experienced an abortion or a stillbirth and included 17,251 RDW with live birth as the study subject. If a woman had twin births, then the last child's information on neonatal complications and care-seeking behavior was collected. Figure 1 shows the study participant selection for the analysis of this paper.

### Data collection and quality control

The data collection instrument was adapted from the Bangladesh Demographic and Health Survey (BDHS) questionnaire (2, 3). The study investigators pre-tested the adapted questionnaire before data collection. We have prepared the tools in both English and Bangla. However, all interviews were conducted in the Bangla language only. The interview questionnaire included information on socio-demographic characteristics of the households, women's background characteristics, childbirth history, health service-related information, maternal health care practices, child's experience of complications, and care-seeking behaviors for sick neonates. We recruited data collectors and supervisors who had extensive experience in survey data collection and most of them had an undergraduate degree in social science. The data collection team comprised 2 field research officers (FRO), 7 field research supervisors (FRS), and 56 data collectors (DC). Each small data collection team consisted of 1 FRS and 8 DC, deployed for the data collection of 2 districts. The FROs supervised all small teams by visiting the team sequentially. All data collectors and supervisors received training on the data collection tools before the start of data collection. Data collection was carried out from April to September 2019. Data was collected on electronic devices, with built-in skips and immediate identification of erroneous entry was built in to improve data quality at the time of entry. FRO and FRS accompanied the DC during data collection. Supervisors organized regular team meetings for supervisory feedback, data rechecking and discussing data collection issues. A quality control (QC) team comprised of interviewers and field-based supervisors monitored and ensured data integrity by re-interviewing and matching with original data. The QC team re-interviewed approximately 3% of the total interviews.

### Hypothesized conceptual/theoretical model

**Figure 2:**
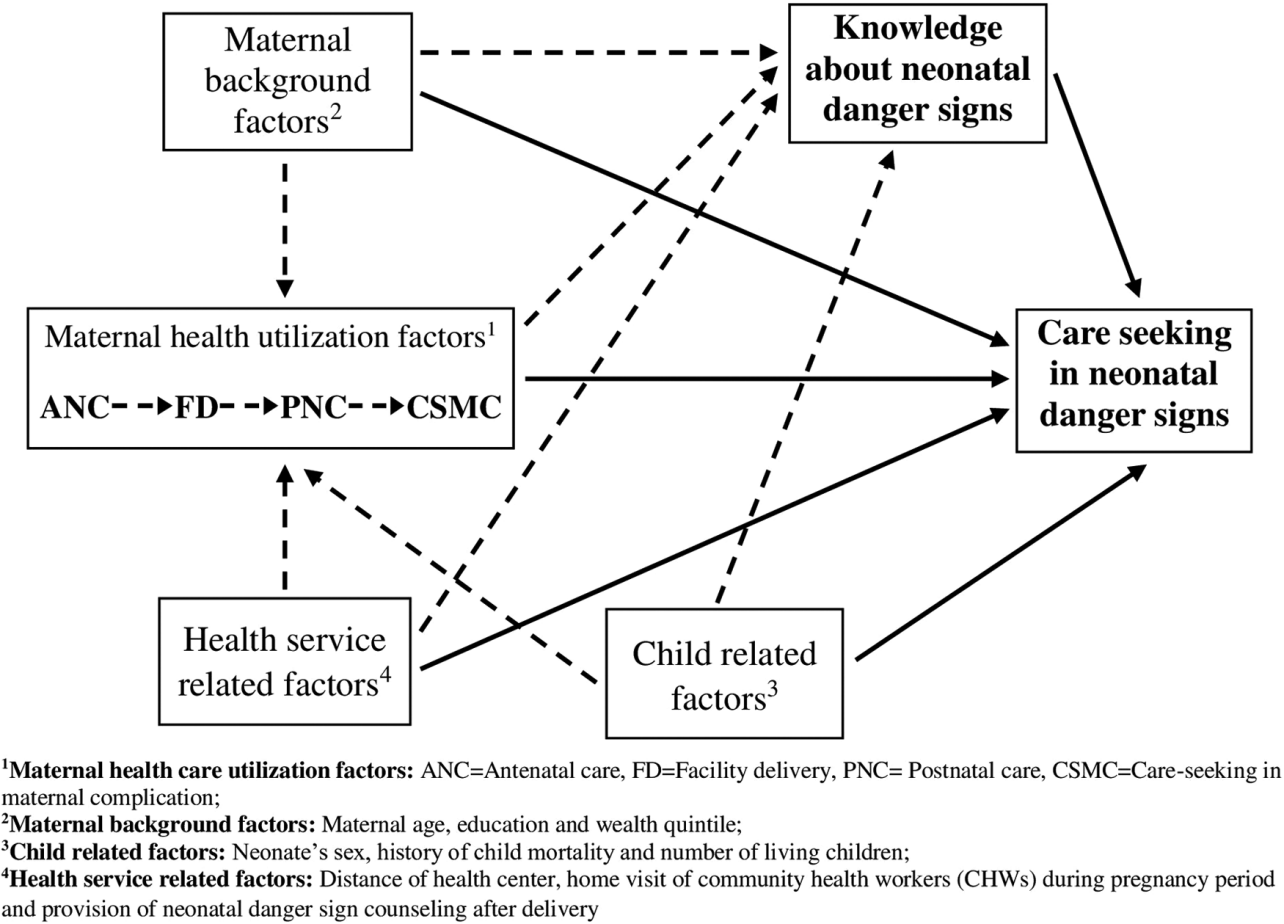
Hypothetical inter-relationship of different factors associated with care-seeking for neonatal danger signs.

Our hypothesized inter-relationship among predictors and main outcome variables was built based on an extensive literature review and the plausibility of inter-relationships (Figure 2). Lines indicate the relationships and direction of relationships between the variables. The lack of a line implies no hypothesized relationship between the variables (34). The variables identified as predictors of care-seeking behaviors for neonatal danger signs were placed into five groups/domains: (1) maternal background characteristics, (2) maternal health care utilization factors, (3) child-related factors, (4) health service-related factors and (5) knowledge on neonatal danger signs. The hypothesized predictors of each domain and scientific evidence used to construct the theoretical model are included in the Supplementary Information S1.

Relationships between factors and the main outcome variable are characterized as either direct or indirect relationships. A solid line indicates a direct relationship between the predictor and care-seeking behavior. Whereas, an indirect relationship, that is the mediated relationship through a second predictor variable (called a mediator variable), is represented by the dotted line.

**Figure 3:**
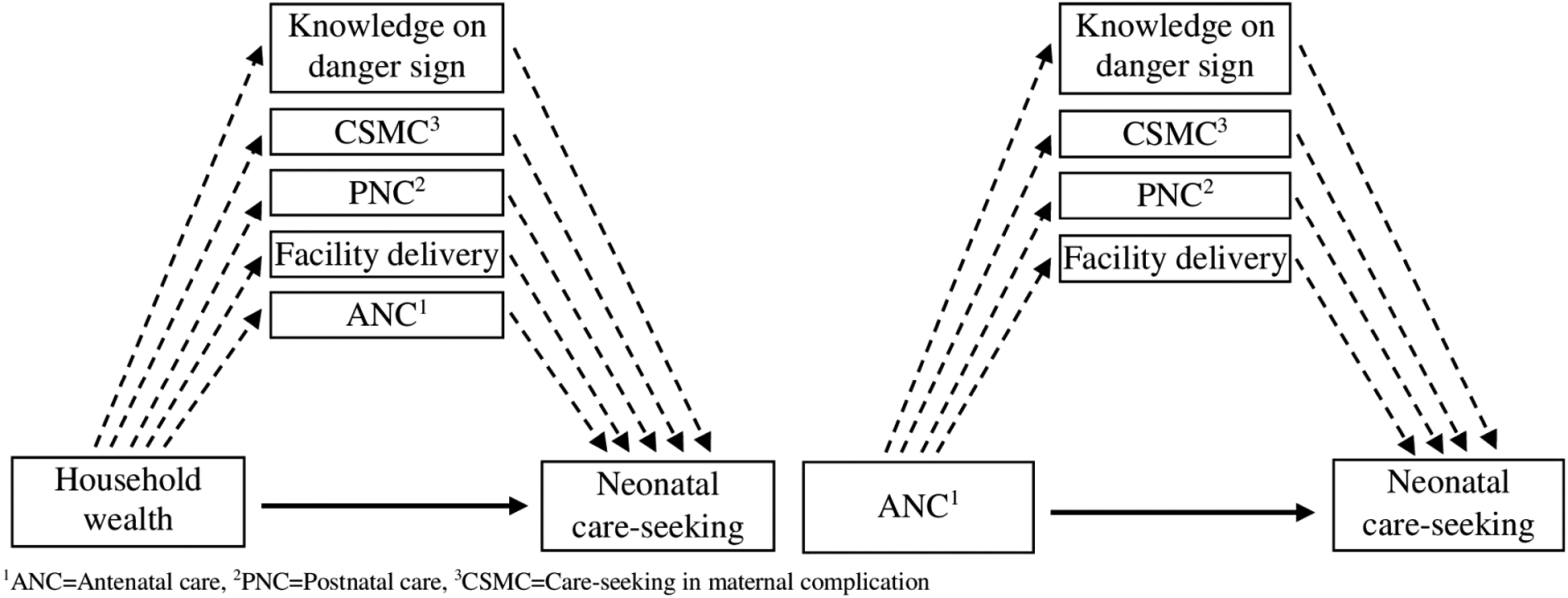
Direct and indirect effect pathway from household wealth and ANC to neonatal care-seeking.

The following Figure 3 is demonstrating the hypothesized direct and indirect effects through mediated pathways for the household wealth and ANC utilization variables. As with Figure 2, the solid represents the direct effect and the dotted arrows show the indirect effects through the mediators.

### Study variables

### Endogenous variables

In the structural equation model, some variables could be simultaneously used as the independent and dependent variables or only the dependent variable. These variables are called endogenous variables. In our GSEM model, six simultaneous multivariable equations of six endogenous/dependent variables were used. The six interrelated endogenous variables were care-seeking for sick neonates from qualified providers, woman's knowledge of neonatal danger signs, care-seeking for last maternal complication from qualified providers, PNC from qualified providers, facility delivery, and attendance of four or more ANC visits from qualified providers. Medical doctors, nurses, midwives, paramedics, family welfare visitors, medical assistants, sub-assistant community medical officers, and community skilled birth attendants were considered qualified providers (2, 3). Maternal complications were defined as any severe headaches with blurry vision, convulsions, high blood pressure, excessive vaginal bleeding, prolonged labor, body parts except the head coming out first, placenta remaining inside, foul-smelling discharge with high fever, and oedema during pregnancy/delivery/after the delivery period. Knowledge of neonatal danger signs was measured as the number of danger signs for which care should be sought. The interview question consisted of 22 danger sign options, which included difficulty or fast breathing, pneumonia, cold/cough, low temperature, high temperature, yellow skin/eye, poor sucking or feeding, discharge from the umbilical cord, skin lesions/blisters, red or swollen eyes with pus, skin rash, measles, convulsions/spasms/rigidity, lethargy/unconsciousness, baby doesn't cry, doesn't pass urine, doesn't pass stool, continuous vomiting, distended abdomen, difficult to wake, diarrhea, and chest in-drawing. Interviewers did not read out the danger signs options while asking this question. For the following question, interviewers read out all the above mentioned danger signs and asked the women whether the child experienced any of these danger signs. Then the subsequent care-seeking behavior was asked. As endogenous variable neonatal danger sign knowledge was used as a continuous variable. All the other endogenous variables were binomial, categorized as yes or no.

### Exogenous variables

All the variables used only as independent variables in the structural model are called exogenous variables. The exogenous variables included in the model were maternal age, education, wealth quintile, the total number of live children, history of child death, sex of newborn, distance to the nearest health facility, facility delivery, home visit by a community health worker (CHW) during the pregnancy period, and receipt of any counseling on neonatal danger signs after delivery. We categorized the distance to the nearest health facility into two categories (<5 km and 5 km or more) and operationally defined “<5 km” as the near distance and “5 km and more” as the long distance (2, 3). For the construction of the household wealth quintile, we used household assets, construction materials of household (roof, walls, and floor), source of drinking water, toilet type, number of rooms in the household, ownership of homestead and agricultural land, ownership of domestic animals (cattle, goat, and chicken) and type of fuel used for cooking. For the exogenous variable on women's knowledge of neonatal danger signs, it was categorized into three categories (0, 1–4, and 5+). We operationally defined the danger sign knowledge of less than 5 as low knowledge of newborn danger signs (86). Consistent with other similar literature, the standard category of other exogenous variables was used in the structured equation model (9, 21, 23, 26, 40–42).

### Data analysis

First, we did a descriptive summary of all maternal background characteristics, child-related factors, health service-related factors, and all maternal and child health utilization factors. The descriptive analysis was also done among healthy neonates and sick neonates. Then, we used GSEM in the subpopulation of sick neonates (*N* = 8765) to estimate the inter-relationships of the predictor variables and newborn care-seeking. In our GSEM model, we used the Bernoulli family and logit link function for binary endogenous variables (4 + ANC from qualified providers, facility delivery, PNC from qualified providers, care-seeking of maternal complication, and care-seeking of sick neonates) and gaussian family and identity link function for the continuous endogenous variable (knowledge on neonatal danger signs) (36). Additionally, the robust standard error was considered as mitigation of the multivariate normality (MVN) assumption (37). The indirect (mediated) effect of each indirect pathway of each mediator was calculated with multiplicated coefficients. The total indirect effect of the variable was estimated by the sum of multiplicated coefficients of all mediated pathways. The total effect of each indicator was calculated as a sum of the direct and total indirect effects. These indirect, direct, and total effects were obtained using the “nlcom” post-estimation command after the GSEM model. All the indirect, direct, and total effects were estimated and reported in terms of Log Odds (LOD), which has the linear additive property. Using LOD calculation of mediating proportion and cross-comparison of effect can be made. The mediation proportion was calculated by dividing the indirect over the total effect (38). Due to the comparability of effects across the predictors, the average relative effect was calculated for the multi-categorical independent variable (39). As a supplementary analysis, we also conducted the distribution of very severe neonatal complications according to neonate's sex (Supplementary Table S1) and the care-seeking distribution of qualified and unqualified providers according to reported very severe complications (Supplementary Table S2). All data analysis was conducted in STATA, version 14.0.

### Ethical consideration

Ethical approval was granted by the Ethical Review Committee of Save the Children USA and the Institutional Review Board (IRB) of icddr,b (Protocol number: PR#18099). Informed written consent was obtained from the study participants after explaining the purpose of the data collection, methods and procedure, use of data, risks, benefits, principle of no compensation, measures for privacy and confidentiality, their voluntary participation, and right to withdraw from the study at any time without showing any cause. For mothers below 18 years, we also took informed written consent from a legal guardian or next of kin.

## Results

### Description of study participants

The majority of mothers were between 20 and 34 years of age (76.9%) and 15.2% of mothers were less than 20 years. About 7.3% of mothers had no education, 12.5% stopped school before completing primary education, and only 21.3% completed secondary or higher levels of study. Overall, 34.7% of mothers reported that this was their first child and 10% experienced a child death in the past.

Overall, less than one-third (27.3%) of mothers received four or more ANC visits from qualified providers during their last pregnancy, around half (52.2%) gave birth in a health facility, 43.4% received PNC from qualified providers, and 65.0% sought care from qualified providers during their most recent maternal complication (*N* = 4713). Mothers' knowledge of neonatal danger signs was low. Out of 22 neonatal danger signs, 3.3% of mothers of sick neonates had no knowledge of any neonatal danger sign, 76.4% knew 1–4 danger signs and only 20.3% of mothers knew 5 or more danger signs (Table 1).

**Table 1:** Distribution of overall background characteristics of the respondents, and disaggregated by the healthy and sick neonates.

Indicators (%)	Overall (*N* = 17,251)	Newborn with no reported illness (*N* = 8486)	Newborn with reported illness^a^ (*N* = 8765)
	*n* (%)	*n* (%)	*n* (%)
**Mother's age**			
<20	2,629 (15.2)	1,281 (15.1)	1,348 (15.4)
20–34	13,263 (76.9)	6,524 (76.9)	6,739 (76.9)
35–49	1,359 (7.9)	681 (8.0)	678 (7.7)
**Mother's education**			
No education	1,267 (7.3)	696 (8.2)	571 (6.5)
Primary incomplete	2,164 (12.5)	1,074 (12.7)	1,090 (12.4)
Primary complete	2,643 (15.3)	1,364 (16.1)	1,279 (14.6)
Secondary incomplete	7,507 (43.5)	3,567 (42.0)	3,940 (45.0)
Secondary complete or higher	3,670 (21.3)	1,785 (21.0)	1,885 (21.5)
**Wealth quintile**			
Lowest	3,319 (19.2)	1,638 (19.3)	1,681 (19.2)
Second	3,353 (19.4)	1,666 (19.6)	1,687 (19.2)
Middle	3,473 (20.1)	1,691 (19.9)	1,782 (20.3)
Fourth	3,519 (20.4)	1,696 (20.0)	1,823 (20.8)
Highest	3,587 (20.8)	1,795 (21.2)	1,792 (20.4)
**Number of living children**			
1	5,992 (34.7)	2,860 (33.7)	3,132 (35.7)
2–3	9,302 (53.9)	4,615 (54.4)	4,687 (53.5)
4+	1,957 (11.3)	1,011 (11.9)	946 (10.8)
**History of child mortality**			
No	15,524 (90.0)	7,644 (90.1)	7,880 (89.9)
Yes	1,727 (10.0)	842 (9.9)	885 (10.1)
**Sex of child**			
Female	8,882 (51.5)	4,603 (54.2)	4,279 (48.8)
Male	8,369 (48.5)	3,883 (45.8)	4,486 (51.2)
**CHW's home visit during ANC**			
No	14,193 (82.3)	7,163 (84.4)	7,030 (80.2)
Yes	3,058 (17.7)	1,323 (15.6)	1,735 (19.8)
**Newborn Danger Signs counseling during ANC**			
No	16,851 (97.7)	8,303 (97.8)	8,548 (97.5)
Yes	400 (2.3)	183 (2.2)	217 (2.5)
**Distance of nearest health facility with delivery care**			
≤5 km	8,146 (47.2)	3,980 (46.9)	4,166 (47.5)
5+ km	9,105 (52.8)	4,506 (53.1)	4,599 (52.5)
**Neonatal danger sign counseling during PNC period**			
No	13,011 (75.4)	6,574 (77.5)	6,437 (73.4)
Yes	4,240 (24.6)	1,912 (22.5)	2,328 (26.6)
**4+ANC from qualified providers**			
No	12,540 (72.7)	6,360 (74.9)	6,180 (70.5)
Yes	4,711 (27.3)	2,126 (25.1)	2,585 (29.5)
**Facility delivery**			
No	8,254 (47.8)	4,147 (48.9)	4,107 (46.9)
Yes	8,997 (52.2)	4,339 (51.1)	4,658 (53.1)
**PNC from qualified providers**			
No	9,765 (56.6)	4,877 (57.5)	4,888 (55.8)
Yes	7,486 (43.4)	3,609 (42.5)	3,877 (44.2)
**Care-seeking for recent maternal complication (*N* = 4713)**			
No	14,188 (35.0)	7,399 (36.8)	6,789 (34.0)
Yes	3,063 (65.0)	1,087 (63.2)	1,976 (66.0)
**Knowledge about the neonatal danger sign**			
0	589 (3.4)	301 (3.5)	288 (3.3)
1–4	13,618 (78.9)	6,919 (81.5)	6,699 (76.4)
5+	3,044 (17.6)	1,266 (14.9)	1,778 (20.3)
**Care-seeking for neonatal danger sign**			
No	—	—	457 (5.2)
Yes	—	—	8,308 (94.8)
**Care-seeking of neonatal danger sign by type of providers**			
No care-seeking			457 (5.2)
Care-seeking from unqualified providers	—	—	5,110 (58.3)
Care-seeking from qualified providers	—	—	3,198 (36.5)

^a^ Neonates reported any of the danger sign in first 30 days of life.

Among all mothers, 8,765 mothers (50.8%) reported that their newborns experienced at least one danger sign in the first month of life. Among the mothers of those sick neonates, almost all (94.4%) sought care from any type of provider, however, only about one-third (36.5%) sought care from a qualified provider (Table 1). Among severe neonatal danger signs, care-seeking from qualified providers was high for difficult or fast breathing (45.4%), chest in-drawing (50.0%), and fever (43.8%) (Supplementary Table S2).

### Identification of direct and indirect predictors and effect pathways on newborn care-seeking

Table 2 details the inter-relationship among maternal health utilization indicators, neonatal danger sign knowledge, maternal background characteristics, child/neonate-related factors, and health service-related factors to explain neonatal care-seeking behavior. In the supplementary materials, we have included a table (Supplementary Table S3) that summarizes the significant inter-relationships of the GSEM model (Table 2), i.e., the significant direct and indirect effect pathway and the effect measures of predictors and mediators on the main outcome variable.

**Table 2:** Analysis of interrelationship among the maternal health utilization factors, maternal background factors, child/neonatal factors, health service factors, neonatal danger sign knowledge and the care-seeking for sick neonates as per hypothetical inter-relationship framework (figure 2) using generalized structural equation modeling (GSEM).

Independent variables	Endogenous/dependent variables in GSEM simultaneous equations					
	4 + ANC from qualified providers (*N* = 8765) AOR^£^ (95% CI)	Facility delivery (*N* = 8765) AOR (95% CI)	PNC from qualified providers (*N* = 8765) AOR (95% CI)	Care-seeking for maternal complication (*N* = 2993) AOR (95% CI)	Knowledge on neonatal danger sign (*N* = 8765) A*β*^¥^ (95% CI)	Care-seeking for sick neonates from qualified providers (*N* = 8765) AOR (95% CI)
**Neonatal danger sign knowledge**						
Knowledge about the neonatal danger sign						
0	—	—	—	—	—	1
1–4	—	—	—	—	—	1.24 (0.96, 1.62)
5+	—	—	—	—	—	1.44* (1.09, 1.90)
**Maternal health care utilization related factors**						
Care-seeking for recent maternal complication						
No	—	—	—	—	—	1
Yes	—	—	—	—	—	1.06 (0.96, 1.18)
PNC from qualified providers						
No	—	—	—	1	Ref	1
Yes	—	—	—	1.96** (1.58, 2.44)	0.46** (0.35, 0.57)	1.18* (1.03, 1.34)
Facility delivery						
No	—	—	1	1	Ref	1
Yes	—	—	41.22** (35.56, 47.77)	1.67** (1.35, 2.07)	−0.35** (−0.46, −0.24)	1.30** (1.14, 1.48)
4 + ANC from qualified providers						
No	—	1	1	1	Ref	1
Yes	—	2.53** (2.27, 2.83)	1.46** (1.27, 1.68)	1.46** (1.20, 1.76)	0.24** (0.15, 0.33)	1.26** (1.14, 1.40)
**Maternal background factors**						
Mother's age						
<20	1	1	1	1	Ref	1
20–34	1.24** (1.06, 1.45)	1.25** (1.07, 1.45)	0.99 (0.81, 1.20)	1.29 (1.00, 1.67)	0.21** (0.10, 0.33)	0.91 (0.79, 1.04)
35–49	1.04 (0.79, 1.36)	1.99** (1.56, 2.53)	1.62** (1.15, 2.26)	1.49 (0.99, 2.22)	0.22* (0.03, 0.41)	1.07 (0.85, 1.34)
Mother's education						
No education	1	1	1	1	Ref	1
Primary incomplete	1.18 (0.89, 1.58)	0.94 (0.75, 1.17)	1.68** (1.23, 2.3)	1.05 (0.73, 1.51)	0.11 (−0.05, 0.28)	0.83 (0.66, 1.03)
Primary complete	1.36* (1.03, 1.79)	1.08 (0.86, 1.34)	1.55** (1.14, 2.1)	0.95 (0.66, 1.35)	0.22** (0.06, 0.39)	1.03 (0.83, 1.28)
Secondary incomplete	1.73** (1.33, 2.24)	1.39** (1.14, 1.7)	1.85** (1.40, 2.45)	1.22 (0.87, 1.71)	0.26** (0.11, 0.42)	0.91 (0.75, 1.11)
Secondary complete or higher	2.62** (2.00, 3.45)	1.88** (1.50, 2.35)	2.22** (1.63, 3.02)	1.27 (0.87, 1.86)	0.47** (0.29, 0.64)	0.85 (0.68, 1.05)
Wealth quintile						
Lowest	1	1	1	1	Ref	1
Second	1.14 (0.95, 1.36)	1.45** (1.25, 1.68)	1.13 (0.92, 1.38)	0.92 (0.71, 1.18)	0.10 (−0.01, 0.22)	1.31** (1.13, 1.52)
Middle	1.30** (1.09, 1.55)	1.90** (1.64, 2.20)	1.11 (0.91, 1.35)	1.15 (0.89, 1.47)	−0.03 (−0.15, 0.08)	1.48** (1.28, 1.73)
Fourth	1.80** (1.52, 2.14)	2.59** (2.22, 3.01)	1.27* (1.04, 1.56)	1.09 (0.84, 1.42)	0.07 (−0.05, 0.19)	1.53** (1.31, 1.78)
Highest	3.40** (2.86, 4.04)	3.52** (2.99, 4.14)	1.39** (1.12, 1.73)	1.00 (0.76, 1.33)	0.08 (−0.05, 0.21)	1.49** (1.27, 1.75)
**Child/newborn related factors**						
Number of living children						
1	1	1	1	1	Ref	1
2–3	0.78** (0.70, 0.88)	0.63** (0.56, 0.70)	1.00 (0.85, 1.16)	1.00 (0.81, 1.23)	0.16** (0.07, 0.25)	1.00 (0.90, 1.12)
4+	0.51** (0.40, 0.64)	0.34** (0.28, 0.41)	0.73* (0.56, 0.95)	1.21 (0.87, 1.66)	0.39** (0.23, 0.54)	0.91 (0.76, 1.10)
History of child mortality						
No	1	1	1	1	Ref	1
Yes	1.30** (1.10, 1.53)	0.93 (0.80, 1.09)	0.97 (0.79, 1.20)	1.08 (0.84, 1.40)	0.01 (−0.11, 0.13)	0.84* (0.72, 0.98)
Sex of newborn						
Female	—	—	—	—	—	1
Male	—	—	—	—	—	1.20** (1.10, 1.31)
**Health service-related factors**						
Distance of nearest health facility with delivery care						
≤5 km	1.25** (1.14, 1.39)	1.12* (1.02, 1.23)	0.97 (0.79, 1.20)	1.08 (0.92, 1.40)	—	1.13** (1.03, 1.23)
5 + km	1	1	1	1		1
CHW's home visit during ANC						
No	1	1	1	1	Ref	1
Yes	2.89** (2.57, 3.25)	0.91 (0.81, 1.03)	1.04 (0.89, 1.22)	1.01 (0.83, 1.23)	−0.03 (−0.13, 0.07)	1.04 (0.93, 1.16)
Received neonatal danger sign counseling after delivery						
No	—	—	—	—	Ref	1
Yes	—	—	—	—	0.12* (0.02, 0.21)	1.12* (1.01, 1.25)

^*^ *p*-value < 0.05.

^**^ *p*-value < 0.01.

^£^ AOR, adjusted odd ratio.

^¥^ Aβ, adjusted beta coefficient of multiple linear regression.

Neonatal care-seeking from a qualified provider was associated with maternal danger sign knowledge. The adjusted odd of neonatal care-seeking behavior was higher among mothers with knowledge of 5 or more neonatal danger signs compared to mothers with no knowledge of neonatal danger signs (AOR: 1.44, 95% CI: 1.09, 1.90). Neonatal care-seeking behavior was not directly associated with maternal care-seeking behavior during complications (AOR: 1.06, 95% CI: 0.96, 1.18).

Three maternal health care utilization indicators (PNC from a qualified provider, facility delivery, and 4 + ANC from a qualified provider) were both directly and indirectly associated with neonatal care-seeking behavior. Mothers who received a PNC visit from a qualified provider were slightly more likely to seek care when their newborn exhibited danger signs (AOR: 1.18, 95% CI: 1.03, 1.34). In addition, maternal PNC also mediated the newborn care-seeking indirectly by increasing the knowledge of neonatal danger signs greatly (Aβ: 0.46, 95% CI: 0.35, 0.57). Among the maternal health utilization factors, health facility delivery directly increased the adjusted odds of care-seeking for neonatal illness (AOR: 1.30, 95% CI: 1.14, 1.48) and indirectly mediated the care-seeking by increasing the odds of receiving a maternal PNC visit (AOR: 41.22, 95% CI: 35.56, 47.77). On the other hand, attending 4 + ANC visits during pregnancy directly increased the odds of neonatal care-seeking behavior of mothers (AOR: 1.26, 95% CI: 1.14, 1.40) and indirectly increased the odds of neonatal care-seeking through increasing the likelihood of receiving a maternal PNC visit (AOR: 1.46, 95% CI: 1.27, 1.68), facility delivery (AOR: 2.53, 95% CI: 2.27, 2.83) and knowledge of neonatal danger signs (Aβ: 0.24, 95% CI: 0.15, 0.33).

Among the maternal background characteristics, only household wealth was directly associated with neonatal care-seeking. The odds of care-seeking for newborn illness increased with household wealth, mothers from households in the higher wealth quintiles were 1.3–1.5 times more likely to seek care than mothers from households in the lowest wealth quintile. Additionally, the wealth quintile indirectly mediated the neonatal care-seeking by increasing the odds of receiving ANC (AOR ranged: 1.30–3.40), facility delivery (AOR ranged: 1.45–3.52), and PNC (AOR ranged: 1.27–1.39). On the other hand, maternal education only indirectly increased the odds of neonatal care-seeking through increasing the knowledge of neonatal danger signs (Aβ ranged: 0.22–0.47), maternal PNC (AOR ranged: 1.68–2.22), facility delivery (AOR ranged: 1.39–1.88) and four or more ANC (AOR ranged: 1.36–2.62) in higher educated groups. Likewise, maternal age also was not associated with neonatal care-seeking behavior directly, however, it significantly increased the neonatal danger sign knowledge consistently (Aβ ranged: 0.22–0.47).

Among the child-related factors, neonatal care-seeking was higher for male children compared to female children (AOR: 1.20, 95% CI: 1.10, 1.31). This could be due to the higher percentage of male children who experienced severe danger signs (54.4%, *p* = 0.001) compared to female children (50.8%); and no difference was seen in qualified care-seeking (*p* = 0.075) during those severe danger signs (Supplementary Table S1). The odds of care-seeking were lower among mothers who had a history of child death (AOR: 0.84, 95% CI: 0.72, 0.97). Having more children significantly decreased the odds of ANC, facility delivery, and PNC, but increased the odds of knowledge of neonatal danger signs.

Among health service indicators, a shorter distance to the nearest health facility (≤5 km) not only directly increased the likelihood of neonatal care-seeking behavior (AOR: 1.13, 95% CI: 1.03, 1.23) but also indirectly increased the odds of neonatal care-seeking by increasing the odds of ANC (AOR: 1.25, 95% CI: 1.14, 1.39) and facility delivery (AOR: 1.12, 95% CI: 1.02, 1.23). Furthermore, receipt of counseling on neonatal danger signs not only directly increased the chance of neonatal care-seeking (AOR: 1.12, 95% CI: 1.01, 1.25) but also indirectly increased neonatal care-seeking by increasing knowledge of neonatal danger signs (Aβ: 0.12, 95% CI: 0.02, 0.21). Having received a CHW home visit during ANC did not impact care-seeking (AOR: 1.04, 95% CI: 0.93, 1.16), though it might have some positive indirect impact on care-seeking by increasing the odds a woman received ANC (AOR: 2.89, 95% CI: 2.57, 3.25).

### Estimating the direct, indirect and total effect of predictor variables on newborn care-seeking

Table 3 shows the decomposition of direct, indirect, and summative total effect (in terms of Log Odds) of the significant inter-related predictors on neonatal care-seeking identified in the above GSEM model (Table 2).

**Table 3:** Indirect effect, direct effect, total effect and mediating proportion of predictors on neonatal care-seeking (mediation analysis result).

Indicators	Indirect effect		Total indirect effect Log Odds (95% CI)	Direct effect Log Odds (95% CI)	Total effect Log Odds (95% CI)	Mediation proportion (%)
	Mediator	Indirect effect Log Odds (95% CI)				
**Maternal health care utilization related factors**						
4 + ANC from qualified providers	Facility delivery	0.24** (0.12, 0.39)	0.40** (0.27, 0.52)	0.23** (0.13, 0.34)	0.63** (0.49, 0.78)	63.5%
	PNC from qualified providers	0.06* (0.01, 0.12)				
	Knowledge on neonatal danger sign	0.09* (0.02, 0.16)				
Facility delivery	PNC from qualified providers	0.60* (0.12, 1.08)	0.47* (0.02, 0.98)	0.26** (0.13, 0.39)	0.74* (0.30, 1.17)	63.5%
	Knowledge on neonatal danger sign	−0.13* (−0.24, −0.02)				
PNC from qualified providers	Knowledge on neonatal danger sign	0.34* (0.07, 0.60)	0.34* (0.07, 0.60)	0.16* (0.03, 0.29)	0.50** (0.21, 0.78)	68.0%
**Maternal background factors**						
Mother's age	Facility delivery	0.12** (0.04, 0.19)	0.20** (0.09, 0.31)	–	0.20** (0.09, 0.31)	–
	Knowledge on neonatal danger sign	0.08* (0.002, 0.16)				
Mother's education	4 + ANC from qualified providers	0.14** (0.05, 0.23)	0.48** (0.32, 0.63)	–	0.48** (0.32, 0.63)	–
	Facility delivery	0.12** (0.04, 0.21)				
	PNC from qualified providers	0.10* (0.01, 0.18)				
	Knowledge on neonatal danger sign	0.12* (0.01, 0.22)				
Wealth quintile	4 + ANC from qualified providers	0.16** (0.08, 0.24)	0.49** (0.37, 0.61)	0.37** (0.25, 0.50)	0.86** (0.70, 1.02)	57.0%
	Facility delivery	0.21** (0.10, 0.32)				
	PNC from qualified providers	0.12* (0.02, 0.22)				
**Child/newborn related factors**						
History of child death	4 + ANC from qualified providers	0.06* (0.01, 0.10)	0.06* (0.01, 0.10)	−018.* (−0.33, −0.02)	−0.12 (−0.28, −0.04)	−50.0%
Number of living children	4 + ANC from qualified providers	−0.11** (−0.17, −0.05)	−0.26** (−0.40, −0.12)	–	−0.26** (−0.40, −0.12)	–
	Facility delivery	−0.21** (−0.31, −0.10)				
	PNC from qualified providers	−0.05 (−0.11, 0.01)				
	Knowledge of neonatal danger sign	0.10* (0.01, 0.19)				
Male sex of newborn	–	–	–	0.18** (0.10, 0.23)	0.18** (0.10, 0.23)	–
**Health service-related factors**						
Lower distance of nearest health facility	4 + ANC from qualified providers	0.05* (0.02, 0.09)	0.08** (0.04, 0.13)	0.12** (0.03, 0.21)	0.20** (0.10, 0.30)	40.0%
	Facility delivery	0.03* (0.002, 0.06)				
CHW's home visit during ANC	4 + ANC from qualified providers	0.24** (0.13, 0.36)	0.24** (0.13, 0.36)	–	0.24** (0.13, 0.36)	–
Received neonatal danger sign counseling after delivery	Knowledge of neonatal danger sign	0.08 (−0.01, 0.18)	0.08 (−0.01, 0.18)	0.11* (0.01, 0.22)	0.20* (0.06, 0.34)	40.0%
**Neonatal danger sign knowledge**						
Neonatal danger sign knowledge	–	–	–	0.37* (0.09, 0.64)	0.37* (0.09, 0.64)	–

^*^ *p*-value < 0.05.

^**^ *p*-value < 0.01.

Among the maternal health care indicators, ANC, facility delivery, and PNC had a direct effect on neonatal care-seeking [ANC::Log Odds (LOD): 0.23, 95% CI: 0.13, 0.34; Facility delivery::LOD: 0.26, 95% CI: 0.13, 0.39; PNC::LOD: 0.16, 95% CI: 0.03, 0.29]. Similarly, ANC, facility delivery and PNC also indirectly increased neonatal care-seeking [ANC::Log Odds (LOD): 0.23, 95% CI: 0.13, 0.34; Facility delivery::LOD: 0.26, 95% CI: 0.13, 0.39; PNC::LOD: 0.16, 95% CI: 0.03, 0.29]. The proportions of indirect effects of ANC, facility delivery and PNC were higher than the direct effect (Mediation proportion range: 63.5%–68.0%). ANC had 3 mediating pathways, and among those, facility delivery was the most proximate to the ANC and mediated the highest indirect effect (LOD: 0.24). Similarly, facility delivery had 2 mediating pathways and the highest indirect effect of facility delivery was mediated through PNC (LOD: 0.60). Finally, all of the indirect effects of PNC were mediated through the women's knowledge of neonatal danger signs (LOD: 0.34). Maternal health utilization factors (ANC, facility delivery, and PNC) had the highest summative total effects (Log Odds (LOD): 1.80) comparing the summative total effect of each maternal background, child/newborn, and health service-related factors.

Among the maternal background factors, household wealth had both direct (LOD: 0.37, 95% CI: 0.25, 0.50) and indirect effects (LOD: 0.49, 95% CI: 0.37, 0.61). This indirect effect was also the highest among all indirect effects and mediated through ANC, facility delivery, and PNC. Household wealth showed the highest total effect (LOD: 0.86, 95% CI: 0.70, 1.02) among all other predictors of neonatal care-seeking. On the other hand, maternal education did not show any direct effect, though it showed the second highest indirect effect among all other predictors of neonatal care-seeking (LOD: 0.48, 95% CI: 0.32, 0.63).

Among the child-related factors, the likelihood of neonatal care-seeking was higher among the male children (LOD: 0.18, 95% CI: 0.10, 0.23), while care-seeking was less among mothers who had a history of child death (LOD: −0.12, 95% CI: −0.28, −0.04). Similarly, neonatal care-seeking was lower among the mothers with more children (LOD: −0.26, 95% CI: −0.40, −0.12).

Among the health service-related indicators, receipt of a CHW visit during pregnancy indirectly increased neonatal care-seeking (LOD: 0.24, 95% CI: 0.13, 0.36). Following the CHW visit, distance to the nearest health facility and receiving counseling on neonatal danger signs after delivery each increased the total log odds of neonatal care-seeking by 0.20 times (95% CI: 0.10, 0.30 and 0.06, 0.34 respectively).

On the other hand, as the most proximal predictor, women's knowledge of neonatal danger signs only directly increased the log odds of neonatal care-seeking (LOD: 0.37, 95% CI: 0.09, 0.64). It was also the significant mediating channel for most other variables of the conceptual framework.

## Discussion

This study found a high prevalence (one of every two neonates) of neonatal danger signs in the first month after birth, which was consistent with the findings of other studies conducted in Bangladesh (9, 26). Although almost all mothers of infants with danger signs sought care, only one-third sought care from a qualified health provider, which was consistent with a recent study in Bangladesh (23). Two-thirds of mothers sought care from untrained providers, which include village doctors, homeopathic practitioners, and local drug stores. In Bangladesh, village doctors are the most frequent choice due to easy access, and because their services are relatively cheap and culturally acceptable (21, 23, 26, 40, 41). In addition, parents believe that homeopathic medicine is mild and has a gentle effect, is easy to administer, and has no side effects, and thus, is considered more suitable for neonates (26, 42). Training of unqualified providers on timely referral to health facilities could be considered to facilitate the appropriate diagnosis and treatment of sick neonates (26).

Among the maternal health utilization factors, ANC, facility delivery, and PNC from qualified providers directly increased neonatal care-seeking from a qualified provider, which was similar to other studies (23, 24, 43, 44). Previous visits to qualified providers increase not only the trust, but also awareness about the availability of newborn care services, and the importance of care-seeking from trained health personnel during an illness episode (23, 26, 45). On the other hand, the indirect effects of ANC, facility delivery and PNC were larger than the direct effect. In conventional determinant analysis, only the direct effects are calculated, which underestimates the overall total effect of a predictor variable on the outcome variable. Therefore, in addition to the direct effect, the greater indirect effect implies the further importance of these factors as the determinants of neonatal care-seeking.

In summary, the highest summative effect of maternal health factors (ANC, facility delivery, and PNC) on newborn care-seeking over other factors domains suggests that ensuring mothers' use of services along the continuum from pregnancy through the postnatal period should be the key programmatic focus of maternal and child health care interventions. For improving the maternal care continuum, some integrated community and health facility-based interventions with the deployment of adequate community-based skilled providers with strong monitoring for continuity were found successful in Bangladesh (46, 47) and other countries (48–50). But further scaling up requires addressing some policy-level challenges, such as recognition of community-based intervention, increasing sustainability and retention of community health workers, addressing the shortage of human resources in public health care, ensuring health workers stay at primary level facilities and innovation in public-private partnership (46, 47, 51). For a resource-poor country like Bangladesh, mobile health (mHealth) strategies have shown promise to improve the timely maternal care continuum (52–54). The national health information management system (HMIS) could be leveraged by integrating a low-cost mHealth innovation within it. Studies also identified the quality of care of maternal health services as an important determinant of the utilization of an adequate number of ANC, facility delivery, and PNC from skilled providers (51, 55–57). Therefore, the quality of maternal care also needs to be prioritized as part of health system strengthening efforts.

Household economic status had the highest total effect on newborn care-seeking, with an almost equal amount of direct and indirect effects; and all indirect effects were mediated through ANC, facility delivery, and PNC. Household income is an enabling factor for care-seeking; and it is obvious that the treatment, medicine, transportation, and opportunity cost for care-seeking are key barriers for poorer households (58–60). In many settings including Bangladesh, demand-side financing (DSF) schemes, such as conditional cash transfers or vouchers; and expansion of services in low-coverage areas for the poorer households have been reported to be effective to reduce inequities for all socioeconomic groups in maternal and child healthcare utilization (61–64). On the other hand, maternal education did not show any direct effect on neonatal care-seeking, which was similar to other study findings (24, 26). However, maternal education did indirectly show a substantial effect on neonatal care-seeking through ANC, facility delivery, PNC, and the mother's knowledge of newborn danger signs. The total indirect effect size of maternal education was large enough to show that maternal education is an important positive factor for improving neonatal care-seeking.

Our study results showed higher care-seeking practices for male newborns compared to females. However, this higher likelihood of care-seeking was explained by the higher number of severe complications among the male neonates, but equal care-seeking proportion during those complications (Supplementary Table S1). Previous studies from Bangladesh have shown a significant sex differential in neonatal care-seeking (21, 24, 26, 65, 66); while our study suggests that there is no apparent gender bias in neonatal care-seeking.

Among the health service-related factors, distance to the nearest health facility was directly and indirectly associated with neonatal care-seeking from qualified providers, which is a similar finding to other studies conducted in Bangladesh (24, 67). Long distance to the nearest health facility is a major barrier to accessing health care services (68–71). The government's standard operating procedure for sick newborns requires referring to Upazila Health Complex (UHC) or District Hospital (DH) (72). At the sub-district level, there is only one UHC (73) and mothers may refuse referral for various reasons including distance to the referral facility, poor roads, or lack of transportation. However, sick newborns with suspected bacterial infections who can't be referred for any reason need to be treated with antibiotics at a primary (Union) level facility (74). The government should encourage and improve local providers' adherence to guidelines for antibiotic treatment for referral-refused mothers (74). Among other health service factors, counseling on neonatal danger signs after delivery, however, did demonstrate a positive effect in increasing woman's knowledge of neonatal danger signs and subsequently increasing the likelihood of care-seeking from qualified providers for newborn illness (24).

In our conceptual model, neonatal danger sign knowledge was the most proximal factor to neonatal care-seeking; having only a direct effect on care-seeking (75–78) and serving as a mediating channel for most factors to affect neonatal care-seeking for illness episodes. Our study results showed low knowledge levels among women about danger signs. The care-seeking from qualified providers was high if mothers perceived the danger signs as severe (49.0%). However, for actual medically identified severe neonatal danger signs (79–83), care-seeking was low (35.6%) (Supplementary Table S2), which implied the wrong judgment about the severity of danger signs (84). This evidence deems the importance of raising maternal awareness about newborn danger signs, most importantly, raising awareness regarding the severity and subsequent qualified care-seeking options. Counseling and health education should be strengthened at the available contact points during the antenatal, delivery, and postnatal periods at the facility and in the community (75, 78).

## Strengths and limitations

To our knowledge this is the first study that assessed the direct, indirect, and total effect of predictor variables in explaining neonatal care-seeking for neonatal illness/danger signs through GSEM and mediation analysis, using a population-based survey with large sample size. However, our study has some limitations. The data used in our study was cross-sectional and therefore the analysis can't confirm the direction of causality between variables. Additionally, SEM/GSEM analysis is based on a hypothetical model with some simultaneous multivariable regression models. Perfect model identification for each simultaneous regression model may not possible due to variables' availability given the nature of the study and dataset, which may lead to omitted variable bias (37, 85).

## Conclusions

Unqualified village practitioners are the first contacts for most of the mothers of sick neonates. Therefore, training the informal providers on the timely referral of sick neonates to health facilities is important. The inter-relationship and highest summative effect of ANC, facility delivery, and PNC on newborn care-seeking suggested that ensuring the maternal care continuum altogether should be the key programmatic focus of maternal and child health care interventions to improve sick newborn care-seeking. Targeted intervention for poorer households and increasing the community health worker's home visits are also required to improve neonatal care-seeking. The effectiveness of danger sign counseling and higher maternal knowledge deemed the importance of danger sign counseling by the health care providers at the available contact points during the antenatal, delivery, and postnatal period at the facility and in the community. In summary, integrated community and health facility-based strategy combined with the innovation of health system strengthening should be the foundation of maternal and newborn health care utilization and saving newborn lives.

## Data Availability

The datasets presented in this article are not readily available because Due to ethical restrictions related to protecting study participants’ privacy and confidentiality, data access is restricted by the Ethical Review Committee of icddr,b. According to the icddr,b data policy (http://www.icddrb.org/policies), interested parties may contact Ms. Armana Ahmed (aahmed@icddrb.org) with further inquiries related to data access. Requests to access the datasets should be directed to Ms. Armana Ahmed (aahmed@icddrb.org).
