# Supplementary material for: Mother's care-seeking behavior for neonatal danger signs from qualified providers in rural Bangladesh: A generalized structural equation modeling and mediation analysis
Source: Front Pediatr. 2023 Jan 4;10:929157. doi: 10.3389/fped.2022.929157 (PMC9846223; doi:10.3389/fped.2022.929157)
Supplement: Supplementary file 1 [file Table1.doc]

**Supplementary Materials**

**S1: Construction of hypothesized conceptual/theoretical model**

The variables identified as predictors of care-seeking behaviors for neonatal danger signs were placed into five groups/domains.

Hypothetical predictors of theoretical model of neonatal care-seeking by domains

| **Domains** | **Predictors** |
| --- | --- |
| Maternal background characteristics | - Maternal age |
|  | - Maternal education |
|  | - Household wealth |
| Maternal health care utilization factors | - Four or more antenatal care visits from qualified providers (ANC) |
|  | - Facility delivery (FD) |
|  | - Postnatal care from qualified providers (PNC) |
|  | - Care-seeking in maternal complication from qualified providers (CSMC) |
| Child related factors | - Neonate’s sex |
|  | - History of child mortality |
|  | - Number of living child |
| Health service related factors | - Distance of health center |
|  | - Home visit of community health workers (CHWs) during pregnancy period |
|  | - Danger sign counseling after delivery |
| Neonatal danger sign knowledge | - Women’s knowledge about neonatal danger sign |

The construction of the theoretical model of care-seeking behavior was supported by the following evidences:

Maternal health utilization factors in maternal health care continuum i.e., antenatal care (ANC), facility delivery (FD), postnatal care (PNC) and care-seeking during maternal complications (CSMC) are associated with their preceding health care utilization factor [1-4]. Maternal health utilization factors are also associated with neonatal danger sign knowledge gaining and subsequent care-seeking [5-12]. Maternal background characteristics, i.e., mother’s age, education and wealth quintile are associated with neonatal care-seeking behavior, knowledge about neonatal danger sign and maternal health utilization behaviors [1, 6, 7, 11, 13, 14]. Level of neonatal danger sign knowledge is associated with the neonatal care-seeking behavior during complications [15-17]. Child related factors, i.e., neonate’s sex, history of child death and number of living child are also associated with neonatal care-seeking behavior, neonatal danger sign knowledge and maternal health utilization behaviors [1, 5, 6, 9, 18]. Health service related factors, i.e., distance of health center, home visit of community health workers and neonatal danger signs counseling are also associated with neonatal care-seeking behavior, neonatal danger sign knowledge and maternal health utilization behaviors [7, 11, 13-15].

**S1** Table: Distribution of severe neonatal danger signs and care-seeking from qualified providers, disaggregated by female and male neonates

| **Severe neonatal danger signs^1^** | **Total (N=8765)** | **Female neonates (N=4279)** | **Male neonates (N=4486)** | **p-value^2^** |
| --- | --- | --- | --- | --- |
| Difficult or Fast breathing | 12.4 | 10.6 | 14.2 | 0.000 |
| Chest in-drawing | 0.5 | 0.3 | 0.6 | 0.020 |
| Fever | 32.6 | 33 | 32.2 | 0.393 |
| Low body temperature | 0.5 | 0.5 | 0.6 | 0.667 |
| Convulsion/Spasms/Rigidity | 1.9 | 1.7 | 2.1 | 0.136 |
| Poor sucking or feeding | 2.6 | 2.6 | 2.7 | 0.918 |
| No spontaneous movement | 2.5 | 1.8 | 3.2 | 0.000 |
| Yellow skin/palm/feet/eye | 7.0 | 6.6 | 7.4 | 0.128 |
| Skin lesions or blisters, pus, redness with swelling | 4.7 | 4.4 | 4.9 | 0.302 |
| Any severe danger signs | 52.6 | 50.8 | 54.4 | 0.001 |
|  | **Total (N=4444)** | **Female neonates (N=2003)** | **Male neonates (N=2441)** | **p-value^3^** |
| Care-seeking from qualified providers during any severe danger signs | 36.0 | 35.0 | 36.9 | 0.075 |

^1^According to different guidelines and validation studies [19-22], we operationally considered these 9 severe neonatal danger signs out of 22 danger signs we collected by our adapted questionnaire.

^2^Chi-square p-values signify the differences of danger sign between female and male neonates.

^3^Chi-square p-values signify the difference of care-seeking between female and male neonates during any severe danger signs.

**S2** Table: Care-seeking from unqualified and qualified providers, according to the severe neonatal danger signs and perceived severity

| **Severe neonatal danger signs^1^** | **No care-seeking (N=457)** | **Care seek from unqualified providers (N=5110)** | **Care seek from qualified providers (N=3198)** | **p-value^2^** |
| --- | --- | --- | --- | --- |
| Difficult or Fast breathing |  |  |  | 0.000 |
| No | 5.5 | 59.2 | 35.2 |  |
| Yes | 2.9 | 51.7 | 45.4 |  |
| Chest in-drawing |  |  |  | 0.166 |
| No | 5.2 | 58.4 | 36.4 |  |
| Yes | 2.4 | 47.6 | 50.0 |  |
| Fever |  |  |  | 0.000 |
| No | 6.0 | 61.1 | 32.9 |  |
| Yes | 3.6 | 52.6 | 43.8 |  |
| Low body temperature |  |  |  | 0.925 |
| No | 5.2 | 58.3 | 36.5 |  |
| Yes | 4.3 | 60.9 | 34.8 |  |
| Convulsion/Spasms/Rigidity |  |  |  | 0.000 |
| No | 5.2 | 58.0 | 36.7 |  |
| Yes | 3.6 | 73.1 | 23.4 |  |
| Poor sucking or feeding |  |  |  | 0.246 |
| No | 5.2 | 58.2 | 36.6 |  |
| Yes | 4.8 | 63.6 | 31.6 |  |
| No spontaneous movement |  |  |  | 0.000 |
| No | 5.3 | 57.9 | 36.8 |  |
| Yes | 3.7 | 73.4 | 22.9 |  |
| Yellow skin/palm/feet/eye |  |  |  | 0.000 |
| No | 5.2 | 57.3 | 37.6 |  |
| Yes | 6.0 | 72.1 | 21.9 |  |
| Skin lesions or blisters, pus, redness with swelling |  |  |  | 0.000 |
| No | 5.2 | 57.6 | 37.1 |  |
| Yes | 4.7 | 72.3 | 23.0 |  |
| Any severe danger signs |  |  |  | 0.000 |
| No | 6.7 | 55.8 | 37.5 |  |
| Yes | 3.9 | 60.5 | 35.6 |  |
| Perceived severity of symptom |  |  |  | 0.000 |
| Not severe | 6.4 | 67.1 | 26.5 |  |
| Severe | 3.7 | 47.2 | 49.0 |  |

^1^According to different guidelines and validation studies [19-22], we operationally considered these 9 severe neonatal danger signs out of 22 danger signs we collected by our adapted questionnaire.

^2^Chi-square p-value signifies the overall differences of no care seeking, care-seeking from unqualified providers and care-seeking from qualified providers according to severe neonatal danger signs and perceived severity.

**S3 T**able: Summary of significant direct and indirect pathways that were influenced the neonatal care-seeking by the predictors and mediators, found in GSEM interrelationship analysis in table 2

| **Pathway of direct effect** | | | **Pathway of indirect effect** | | | | | |
| --- | --- | --- | --- | --- | --- | --- | --- | --- |
| **Predictors to Outcome** | | **AOR**^£^ | **Predictors to Mediator** | | **AOR/Aβ**^¥^ | **Mediator to outcome** | | **AOR** |
| **Maternal health care utilization related factors** | | | | | | | | |
| ANC | Neonatal care-seeking | 1.26 | ANC | Facility delivery | 2.53 | Facility delivery | Neonatal care-seeking | 1.30 |
|  |  |  | ANC | PNC | 1.46 | PNC | Neonatal care-seeking | 1.18 |
|  |  |  | ANC | Neonatal danger sign knowledge | 0.24^¥^ | Neonatal danger sign knowledge | Neonatal care-seeking | 1.44 |
| Facility delivery | Neonatal care-seeking | 1.30 | Facility delivery | PNC | 41.22 | PNC | Neonatal care-seeking | 1.18 |
|  |  |  | Facility delivery | Neonatal danger sign knowledge | -0.35^¥^ | Neonatal danger sign knowledge | Neonatal care-seeking | 1.44 |
| PNC | Neonatal care-seeking | 1.18 | PNC | Neonatal danger sign knowledge | 0.46^¥^ | Neonatal danger sign knowledge | Neonatal care-seeking | 1.44 |
| **Maternal background factors** | | | | | | | | |
| Mother's age | Neonatal care-seeking | --- | Mother's age | Facility delivery | 1.25-1.99^§^ | Facility delivery | Neonatal care-seeking | 1.30 |
|  |  |  | Mother's age | Neonatal danger sign knowledge | 0.21-0.22^§¥^ | Neonatal danger sign knowledge | Neonatal care-seeking | 1.44 |
| Mother's education | Neonatal care-seeking | --- | Mother's education | ANC | 1.36-2.62^§^ | ANC | Neonatal care-seeking | 1.26 |
|  |  |  | Mother's education | Facility delivery | 1.39-1.88^§^ | Facility delivery | Neonatal care-seeking | 1.30 |
|  |  |  | Mother's education | PNC | 1.68-2.22^§^ | PNC | Neonatal care-seeking | 1.18 |
|  |  |  | Mother's education | Neonatal danger sign knowledge | 0.22-0.47^§¥^ | Neonatal danger sign knowledge | Neonatal care-seeking | 1.44 |
| Wealth quintile | Neonatal care-seeking | 1.31-1.49^§^ | Wealth quintile | ANC | 1.30-3.40^§^ | ANC | Neonatal care-seeking | 1.26 |
|  |  |  | Wealth quintile | Facility delivery | 1.45-3.52^§^ | Facility delivery | Neonatal care-seeking | 1.30 |
|  |  |  | Wealth quintile | PNC | 1.27-1.39^§^ | PNC | Neonatal care-seeking | 1.18 |
| **Child/newborn related factors** | | | | | | | | |
| History of child mortality | Neonatal care-seeking | 0.84 | History of child death | ANC | 1.30 | ANC | Neonatal care-seeking | 1.26 |
| Number of living child | Neonatal care-seeking | --- | Number of living child | ANC | 0.51-0.78^§^ | ANC | Neonatal care-seeking | 1.26 |
|  |  |  | Number of living child | Facility delivery | 0.34-0.63^§^ | Facility delivery | Neonatal care-seeking | 1.30 |
|  |  |  | Number of living child | PNC | 0.73 | PNC | Neonatal care-seeking | 1.18 |
|  |  |  | Number of living child | Neonatal danger sign knowledge | 0.16-0.39^§¥^ | Neonatal danger sign knowledge | Neonatal care-seeking | 1.44 |
| Male sex of newborn | Neonatal care-seeking | 1.20 | **---** | **---** | **---** | **---** | **---** | **---** |
| **Health service related factors** | | | | | | | | |
| Health facility distance | Neonatal care-seeking | 1.13 | Health facility distance | ANC | 1.25 | ANC | Neonatal care-seeking | 1.26 |
|  |  |  | Health facility distance | Facility delivery | 1.12 | Facility delivery | Neonatal care-seeking | 1.30 |
| CHW visit | Neonatal care-seeking | --- | CHW visit | ANC | 2.89 | ANC | Neonatal care-seeking | 1.26 |
| Neonatal danger sign counseling | Neonatal care-seeking | 1.12 | Neonatal danger sign counseling | Neonatal danger sign knowledge | 0.12^¥^ | Neonatal danger sign knowledge | Neonatal care-seeking | 1.44 |
| **Neonatal danger sign knowledge** | | | | | | | | |
| Neonatal danger sign knowledge | Neonatal care-seeking | 1.44 | **---** | **---** | **---** | **---** | **---** | **---** |

^£^AOR=adjusted odd ratio, ^¥^Aβ=adjusted beta coefficient of multiple linear regression, ^§^Range of significant AOR/Aβ at different category of predictor

**References (Supplementary materials)**

1. Khanam R, Creanga AA, Koffi AK, Mitra DK, Mahmud A, Begum N, et al. Patterns and determinants of care-seeking for antepartum and intrapartum complications in rural Bangladesh: results from a cohort study. PLoS One. 2016;11(12):e0167814.

2. Chakraborty N, Islam MA, Chowdhury RI, Bari W. Utilisation of postnatal care in Bangladesh: evidence from a longitudinal study. Health & social care in the community. 2002;10(6):492-502.

3. Mohan D, Gupta S, LeFevre A, Bazant E, Killewo J, Baqui AH. Determinants of postnatal care use at health facilities in rural Tanzania: multilevel analysis of a household survey. BMC pregnancy and childbirth. 2015;15(1):1-10.

4. Kamal SM, Hassan CH, Alam GM. Determinants of institutional delivery among women in Bangladesh. Asia Pacific Journal of Public Health. 2015;27(2):NP1372-NP88.

5. Chowdhury HR, Thompson SC, Ali M, Alam N, Yunus M, Streatfield PK. Care seeking for fatal illness episodes in neonates: a population-based study in rural Bangladesh. BMC pediatrics. 2011;11(1):1-8.

6. Chowdhury SK, Billah SM, Arifeen SE, Hoque DME. Care-seeking practices for sick neonates: Findings from cross-sectional survey in 14 rural sub-districts of Bangladesh. PloS one. 2018;13(9):e0204902.

7. Shah R, Mullany LC, Darmstadt GL, Talukder RR, Rahman SM, Mannan I, et al. Determinants and pattern of care seeking for preterm newborns in a rural Bangladeshi cohort. BMC health services research. 2014;14(1):1-12.

8. Nonyane BA, Kazmi N, Koffi AK, Begum N, Ahmed S, Baqui AH, et al. Factors associated with delay in care–seeking for fatal neonatal illness in the Sylhet district of Bangladesh: results from a verbal and social autopsy study. Journal of global health. 2016;6(1).

9. Ahmed S, Sobhan F, Islam A. Neonatal morbidity and care‐seeking behaviour in rural Bangladesh. Journal of tropical pediatrics. 2001;47(2):98-105.

10. Yadeta TA. Antenatal care utilization increase the odds of women knowledge on neonatal danger sign: a community-based study, eastern Ethiopia. BMC research notes. 2018;11(1):1-5.

11. Jemberia MM, Berhe ET, Mirkena HB, Gishen DM, Tegegne AE, Reta MA. Low level of knowledge about neonatal danger signs and its associated factors among postnatal mothers attending at Woldia general hospital, Ethiopia. Maternal health, neonatology and perinatology. 2018;4(1):1-8.

12. Ahmed S, Sobhan F, Islam A. Neonatal morbidity and care-seeking behaviour in rural areas of Bangladesh: International Centre for Diarrhoeal Diseases Research Bangladesh: Dhaka; 1998.

13. Sikder SS, Labrique AB, Craig IM, Wakil MA, Shamim AA, Ali H, et al. Patterns and determinants of care seeking for obstetric complications in rural northwest Bangladesh: analysis from a prospective cohort study. BMC health services research. 2015;15(1):1-13.

14. Bulto GA, Fekene DB, Moti BE, Demissie GA, Daka KB. Knowledge of neonatal danger signs, care seeking practice and associated factors among postpartum mothers at public health facilities in Ambo town, Central Ethiopia. BMC research notes. 2019;12(1):1-7.

15. Bogale TN, Worku AG, Yalew AW, Bikis GA, Tigabu Kebede Z. Mothers treatment seeking intention for neonatal danger signs in Northwest Ethiopia: a structural equation modeling. Plos one. 2018;13(12):e0209959.

16. Ekwochi U, Ndu IK, Osuorah CD, Amadi OF, Okeke IB, Obuoha E, et al. Knowledge of danger signs in newborns and health seeking practices of mothers and care givers in Enugu state, South-East Nigeria. Italian journal of pediatrics. 2015;41(1):1-7.

17. Ekwochi U, Ndu IK, Osuorah CD, Onah KS, Obuoha E, Odetunde OI, et al. Delays in healthcare delivery to sick neonates in Enugu South-East Nigeria: an analysis of causes and effects. Journal of Public Health. 2016;38(2):e171-e7.

18. Ismail SA, McCullough A, Guo S, Sharkey A, Harma S, Rutter P. Gender-related differences in care-seeking behaviour for newborns: a systematic review of the evidence in South Asia. BMJ global health. 2019;4(3):e001309.

19. MOHFW. National neonatal health strategy and guidelines for Bangladesh. Ministry of Health and Family Welfare, Government of the People's Republic of Bnagladessh; 2009.

20. WHO. WHO recommendations on postnatal care of the mother and newborn: World Health Organization; 2014.

21. Baqui AH, Arifeen SE, Rosen HE, Mannan I, Rahman SM, Al‐Mahmud AB, et al. Community‐based validation of assessment of newborn illnesses by trained community health workers in Sylhet district of Bangladesh. Tropical Medicine & International Health. 2009;14(12):1448-56.

22. Darmstadt GL, Baqui AH, Choi Y, Bari S, Rahman SM, Mannan I, et al. Validation of a clinical algorithm to identify neonates with severe illness during routine household visits in rural Bangladesh. Archives of disease in childhood. 2011;96(12):1140-6.
